# Ecthyma gangrenosum

**DOI:** 10.11604/pamj.2018.30.95.6244

**Published:** 2018-06-05

**Authors:** Ahlam Abdou, Badredine Hassam

**Affiliations:** 1 University Mohammed V, Ibn Sina, Hospital University, Rabat, Morocco

**Keywords:** Pseudomonas, ecthyma, enfant, Pseudomonas, ecthyma, child

## Abstract

Ecthyma gangrenosum (EG) is a severe potentially lethal cutaneous infection that progresses sequentially from maculopapular rash to haemorrhagic bulla and then to necrotic ulceration with surrounding erythema. It usually occurs in immunocompromised patients (aplasia secondary to chemotherapy, HIV infection, neutropenia or functional deficit of neutrophils, agammaglobulinemia). It rarely affects healthy people. Differential diagnosis includes leishmaniasis, pyoderma gangrenosum, eschars and papulonecrotic tuberculides. Blood cultures and/or local sample allow the isolation of P. aeruginosa, which is the causative germ. Treatment is based on suitable parenteral antibiotic therapy according to results of susceptibility tests (third-generation cephalosporins, fluoroquinolones). We here report the case of a 2-year old child with no particular past medical history presenting with multiple necrotic ulcers on the back with erythematous border evolving in a febrile context. Blood culture was negative. Laboratory tests showed sedimentation rate 30 mm, CRP 80mg/l. Bacteriological sampling of pus revealed pseudomonas aeruginosa. The diagnosis of ecthyma gangrenosum was made. The patient underwent parenteral third-generation cephalosporins. Complete patient recovery with healing occurred after 4 weeks.

## Image en médecine

Ecthyma gangrenosum (EG) est une infection cutanée sévère, potentiellement mortelle qui évolue séquentiellement d’abord à type d’éruption maculo-papuleuse, puis une bulle hémorragique, puis vers une ulcération nécrotique entourée d’ un érythème EG se produit généralement chez les patients immunodéprimés (aplasie secondaire à la chimiothérapie, infection par le VIH, neutropénie ou déficit fonctionnel des polynucléaires neutrophiles, agammaglobulinémie). Rarement, il affecte les individus en bonne santé. Le diagnostic différentiel inclut la leishmaniose, le pyoderma gangrénosum, les escarres et les tuberculides papulo-nécrotiques. Les hémocultures et/ou le prélèvement local permettent d’isoler le germe en cause qui est toujours un *P. aeruginosa*. Le traitement repose en une antibiothérapie parentérale adaptée à l’antibiogramme (céphalosporine de troisième génération, fluoroquinolones). Nous présentons le cas d’un enfant de 2 ans sans antécédent pathologiques notables qui a présenté de multiple ulcérations nécrotiques localisées au niveau du dos avec une bordure érythémateuse le tout évoluant dans un contexte fébrile. L’hémoculture n’a pas mis en évidence de germe. Le bilan biologique a objectivé une VS élevée à 30 mm, une CRP à 80mg/l. Un prélèvement bactériologique du pus a révélé le pseudomonas aeruginosa. Le diagnostic d’ecthyma gangrénosum a été posé. Le patient a été mis sous céphosporine troisième génération par voie parentéale. La guérison complète avec cicatrisation a eu lieu après 4 semaines.

**Figure 1:**
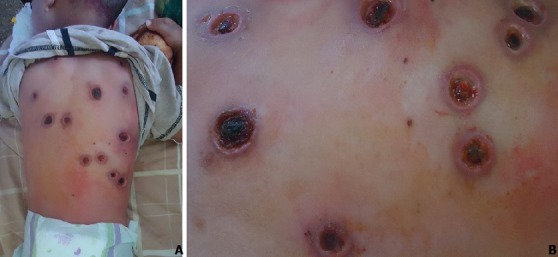
A) multiples ulcérations nécrotiques du dos; B) ulcérations nécrotiques

